# Open-tES: An open-source stimulator for transcranial electrical stimulation designed for rodent research

**DOI:** 10.1371/journal.pone.0236061

**Published:** 2020-07-14

**Authors:** Solène Pedron, Stéphanie Dumontoy, Julien Dimauro, Emmanuel Haffen, Patrice Andrieu, Vincent Van Waes

**Affiliations:** Laboratory of Integrative and Clinical Neuroscience EA481, Université Bourgogne Franche-Comté, Besançon, France; Universidade do Estado do Rio de Janeiro, BRAZIL

## Abstract

Non-invasive neuromodulatory techniques, including transcranial direct current stimulation (tDCS), have been shown to modulate neuronal function and are used both in cognitive neuroscience and to treat neuropsychiatric conditions. In this context, animal models provide a powerful tool to identify the neurobiological mechanisms of action of tDCS. However, finding a current generator that is easily usable and which allows a wide range of stimulation parameters can be difficult and/or expensive. Here, we introduce the Open-tES device, a project under a Creative Commons License (CC BY, SA 4.0) shared on the collaborative platform Git-Hub. This current generator allows tDCS (and other kinds of stimulations) to be realized, is suitable for rodents, is easy to use, and is low-cost. Characterization has been performed to measure the precision and accuracy of the current delivered. We also aimed to compare its effects with a commercial stimulator used in clinical trials (DC-Stimulator Plus, NeuroConn, Germany). To achieve this, a behavioral study was conducted to evaluate its efficacy for decreasing depression related-behavior in mice. The stimulator precision and accuracy were better than 250 nA and 25 nA, respectively. The behavioral evaluation performed in mice in the present study did not reveal any significant differences between the commercial stimulator used in clinical trials and the Open-tES device. Accuracy and precision of the stimulator ensure high repeatability of the stimulations. This current generator constitutes a reliable and inexpensive tool that is useful for preclinical studies in the field of non-invasive electrical brain stimulation.

## Introduction

Non-invasive neuromodulatory techniques have beneficial effects on several psychiatric disorders (e.g. depression and addiction [1–5]) and improve the performance of a variety of cognitive domains [6]. They also have been studied as a neurorehabilitation strategy and showed great promise for improving patient conditions in various neurological disorders (see review [7]), such as stroke [8], epilepsy ([9]; including pediatric populations [10]) or neurodegenerative diseases [11]. Finally, these techniques have been studied for their potential capacity to relieve pain in several disorders such as migraine [12], multiple sclerosis [13], neuropathic pain [14,15] or fibromyalgia syndrome [16]. Non-invasive neuromodulatory techniques include various forms of low-intensity transcranial electrical stimulation (tES), with transcranial direct current stimulation (tDCS) being the most studied. tDCS uses a constant current (from 1 to 2 mA in human subjects [17]) applied directly to the head, which modulates cortical excitability for more than 1 hour [18]. Briefly, cathodal stimulation hyperpolarizes, while anodal stimulation depolarizes the resting membrane potential (without triggering action potentials: sub-threshold effect), although the effects depend on various parameters such as the duration and intensity of the stimulation [19–21]. During transcranial alternating current stimulation (tACS) or random noise stimulation (tRNS), electrical frequencies are applied to interfere with intrinsic cortical oscillations. These kinds of stimulations can also be blended with tDCS, e.g. oscillatory tDCS (otDCS). With appropriate parameters, they produce neuroplastic effects [22]. Despite accumulating evidence supporting the efficacy of tES as a treatment options in clinical practice, the neurobiological mechanisms underlying their beneficial effects remain poorly understood. Animal models (mostly rodent models) are used to investigate these mechanisms, validate the safety of the techniques, and optimize the parameters of stimulation (e.g. [4,5,23–26]).

In most cases, tES is applied using commercial stimulators [5,27,28]. For example, the DC-Stimulator Plus from NeuroConn (Germany) allows an adjustable current in 25 μA increments. This kind of stimulator is highly adapted to clinical use but may not be sufficiently precise for rodent studies.

Another option is a Stimulus Isolator, e.g. the A395 Linear Stimulus 25 Isolator (World Precision Instruments, USA) [29,30]. The resolution of these current suppliers is well adapted to rodent studies. However, the current tuning has to be performed manually (from the beginning of the fade-in to the end of the fade-out) or via an input voltage drive. In this case, a waveform generator is required.

The last option is to design a custom-made stimulator. Several teams developed their devices but did not describe them [25,31–33]. For example, Kouzani and collaborators presented a low-cost, programmable, and portable tDCS device adapted for human research [34].

Here, we introduce the Open-tES device, a current generator that is highly suitable for rodent studies, is easy to use, and versatile. This stimulator is low-cost and allows tDCS and other kinds of stimulations. Moreover, our device is shared under a Creative Commons license (CC BY, SA 4.0) and is available on the collaborative platform GitHub (www.github.com/Open-tES).

## Materials and methods

### Physical description

**Fig 1:**
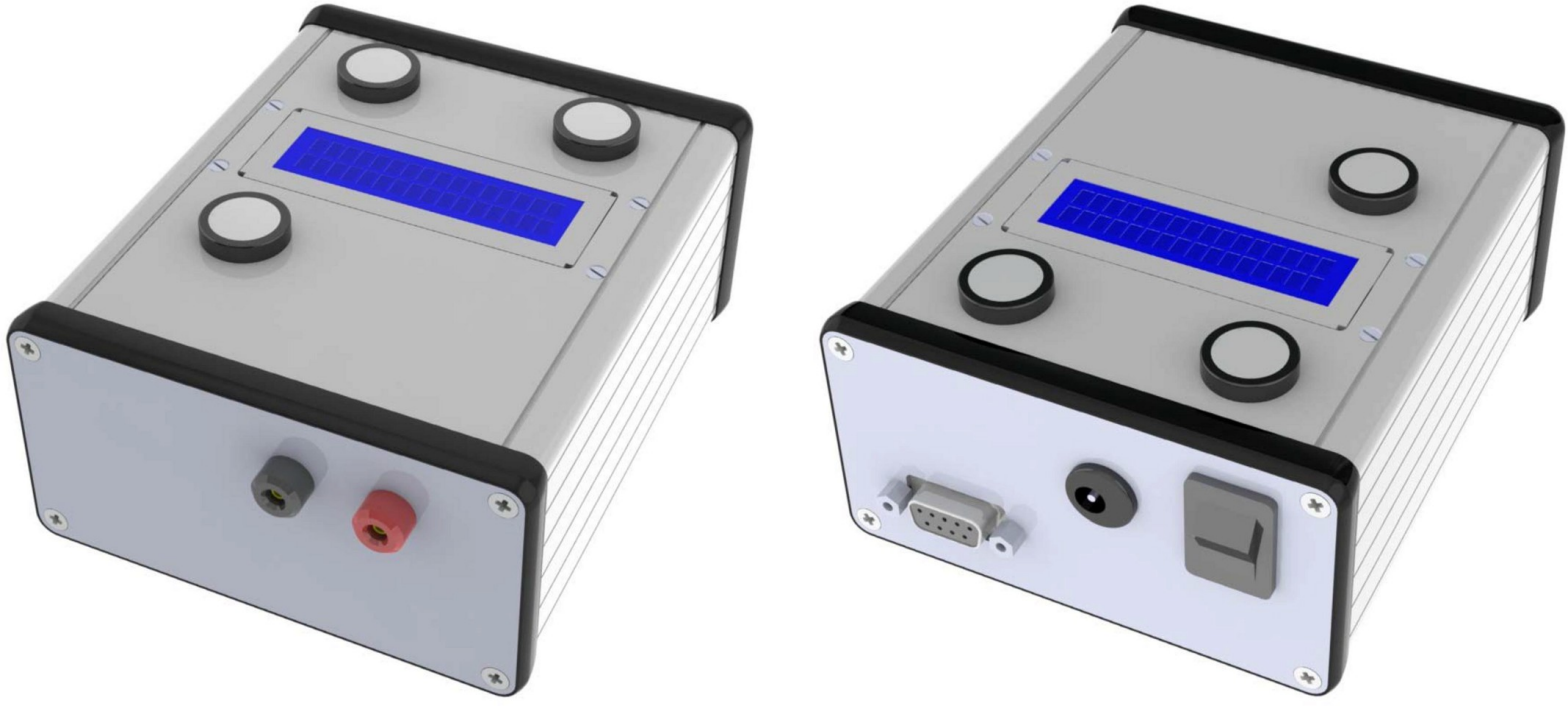
Open-tES stimulator overview. The front panel on the left side and the rear panel on the right side.

The stimulator is enclosed in an aluminum box 120 mm long, 103 mm wide, and 53 mm high (Fig 1). Banana connectors for the anode and cathode are on the front panel. An LCD screen and three push buttons on the top panel allow the stimulator settings to be chosen. The power supply, power switch and serial port are on the rear panel.

### Operating

#### Standalone mode

For tDCS stimulation, parameters are set directly on the device:

- Current (ranges from 10 to 250 μA, increments of 1 μA)
- Duration (ranges from 10 to 3600 s, increments of 10 s)
- Fade-in and fade-out duration (from 2 to 60 s, increments of 1 s)
- Impedance threshold: if the impedance is higher than the user-defined threshold, a warning alerts the user.
- RS232 can be enabled for acquiring impedance on a computer in real-time.
- Current, voltage, impedance, and time remaining until the end of the stimulation are displayed during the stimulation. The stimulation can be suspended with a fade-out. When the fade-out is finished, the stimulation can be aborted or resumed for the remaining time.

#### Computer-controlled mode

For tDCS variants (otDCS, tPCS) or external triggering, a computer-control is required. The current is driven by the computer using an RS232 protocol. Moreover, this protocol allows feedback recording for the voltage and current delivered. As in the stand-alone mode, the current, voltage, impedance, and duration information are displayed on the screen of the stimulator.

#### Electronic design

**Fig 2:**
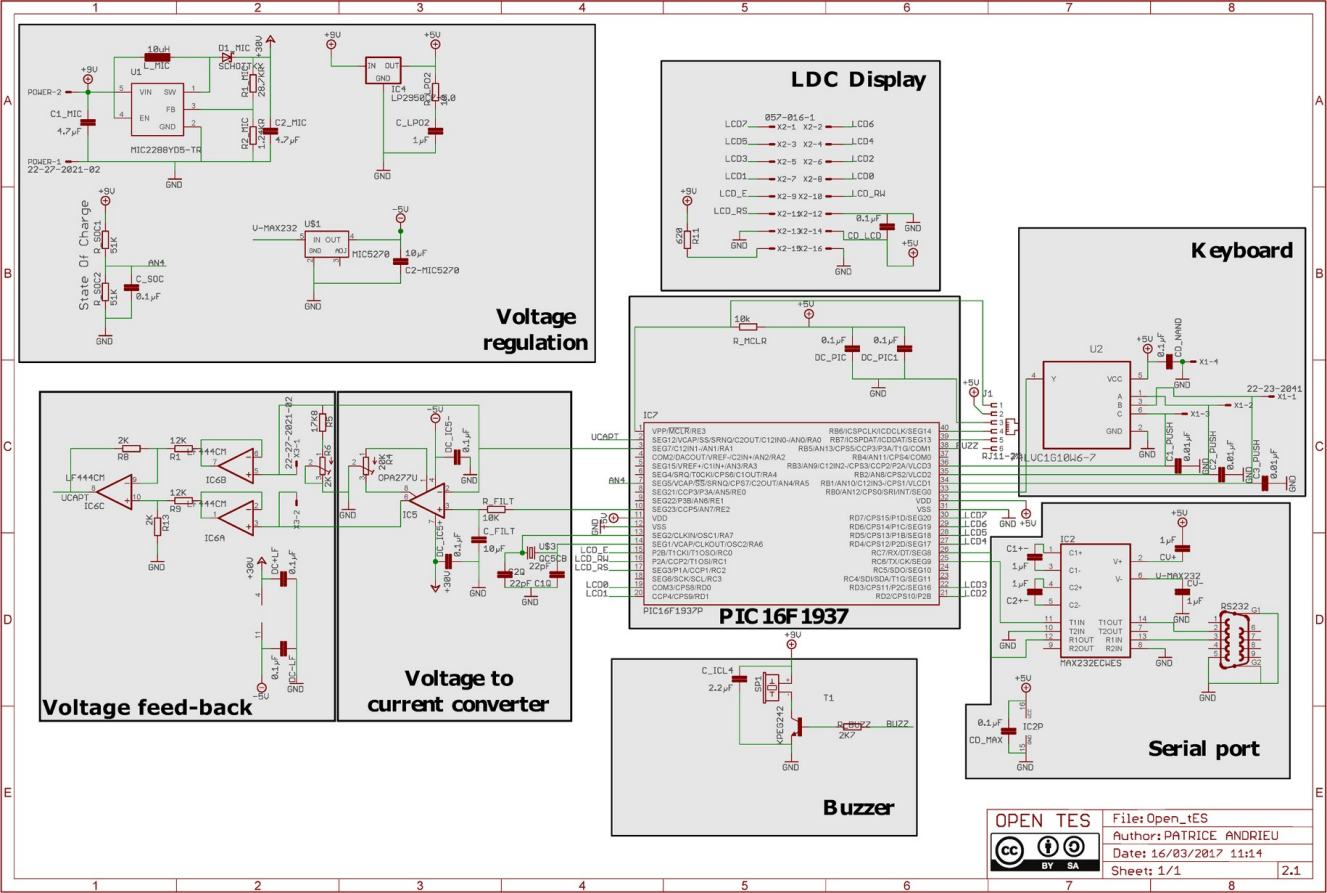
Electrical circuit diagram. Gray boxes group the components by function to facilitate the diagram reading.

Eagle software (Autodesk, CA, USA) has been used to design the printed circuit board. In this section, we briefly describe the circuit. We would recommend consulting Fig 2 while reading the following sections.

#### Microcontroller

The main element of the Open-tES device is the PIC16F1937 microcontroller (Microchip Technology, Arizona, USA). Its operating speed is 32 MHz, which offers a high computational capacity and a high pulse width modulation (PWM) switching frequency.

#### Voltage regulation

The device is powered by an external energy source (power supply, 9V rechargeable battery or 9V battery). The voltage is lowered by a regulator from 9 V to 5 V for supplying digital components. Buzzer and display backlight are directly connected to the energy source. For the Op-amps, the negative power supply is assumed by the MAX232 negative output and regulated with the MIC5270 negative regulator. The positive power supply is generated with the MIC2288 boost converter. With the MIC2288, the voltage output can reach 30 V.

#### Autonomy

An open-tES device consumes around 50 mA. The rechargeable battery lasts for up to 5 hours (NiMH rechargeable battery 9V-Block E Typ 300, Ansmann, Germany), while the 9V battery lasts for up to 24 hours (U9VL-J-P, Ultra life corporation, USA). To check the battery life, voltage is determined through the resistor R_SOC2 with the analog pin AN4. The battery life is displayed on the LCD screen before the start of the stimulation with “BAT OK” or “LOW BAT” as indicators. If “LOW BAT” is indicated, the battery must be replaced before starting the stimulation.

#### Display

Data are transferred between the microcontroller and the display by the microcontroller PORTD (8-bit). Three PORTC pins are used for the transfer control. The resistor R11 limits the current through the backlight display.

#### Keyboard

Button-press grounds the bit 1, 2 or 3 of the PORTB, which is configured in pull up. The CX PUSH capacitors clean up switch bounces. Actions on the keyboard are detected by the NAND IC3C, which is connected to RB0. RB0 is configured as an external interrupt.

#### Buzzer

The microcontroller pin RB5 drives the buzzer via the NPN transistor T1. The resistor R BUZZ limits the base current.

#### Serial port

The communication between the stimulator and the computer is assumed by the Universal Synchronous/Asynchronous Receiver/Transmitter (USART) module of the microcontroller. A MAX232 is used to adapt voltages between both components.

#### Voltage to current converter

The PWM signal is filtered with a 4.5 Hz low-pass filter composed of R_FILT and C_FILT. The filtered signal is applied to the non-inverting input of the op-amp IC5. Negative feedback produces a current I_load_ through the load (the rodent) connected to the X3 connector as follows:

$$I_{load}=\frac{{\text{V}}_{\text{PWM}}}{\left(\text{R}5+\text{R}6\right)}$$

The tension of the PWM ranges from 0 to 5 V, and the resolution is 10 bit. To simplify the microcontroller programming and optimize the processing, the conversion between the digital value and the current must be 1 for 0.25 μA. The trimmer R6 allows the conversion to be adjusted.

#### Voltage and current acquiring

The voltage applied to the op-amp non-inverting input is acquired by the microcontroller via the pin AN1 which is configured as an analog input. This feedback makes sure the tension delivered by the PWM is correct. The AN0 input acquires the voltage across the rodent. The differential amplifier composed by the op-amp IC6C and the resistors R1, R8, R9, and R13 divides the voltage by six (to low it under 5 V) and produces an output referring to the ground. High input impedance is assured by the op-amp buffers IC6A and IC6B.

### Programming of the microcontroller

The stimulator firmware is written in μC with the MPLAB X IDE software and compiled using the MPLAB XC8 C Compiler (Microchip Technology, Arizona, USA). In this section, we quickly describe the structure and key elements of the firmware. For further details, the source code is available on www.github.com/Open-tES.

#### Program structure

The program consists of the main source file and two libraries. One library is dedicated to controlling the display while the second is focused on diverse functions. The configuration of the microcontroller is described in the first part of the main file. In the second part, the variables used by the functions \main" and \interrupt" are declared. Then, the interrupt function and the main function with an infinity loop are written.

#### Keyboard

Pressing on the push-button generates an interruption from RB0. During the interruption, the PORTB is snapped in the variable. Then, the information is processed in an infinity loop. During the process, the keyboard is locked.

#### Fade-in/fade-out

During the fade-in or fade-out, the intensity increases or decreases in increments of 0.25 μA. Therefore, the fade-in/out duration is set by modifying the sample-rate. Here, TIMER4 manages the sample-rate during this period.

#### Stimulation duration

The stimulation duration (in seconds) is written in a variable (integer) before the stimulation starts. After the fade-in, this variable is reduced every second (this clock is generated with TIMER1). When the stimulation is finished, the fade-out starts.

#### Display control

The characters are written on a buffer, which is transferred to the display when necessary. The transfer is operated in an infinity loop, with one character for each iteration, to leave computational time.

#### Display refresh

Every 250 ms (this clock is generated with TIMER6), the channel AN0 is selected to acquire the voltage across the rodent, and the conversion is started. The end of the conversion generates an interruption. Then, the channel AN1 is selected to measure the current, and the conversion is started. A new interruption is generated, during which a flag is positioned to launch the calculation of voltage, current and impedance in an infinity loop. When this operation is done, the display is refreshed with new data.

#### RS232 management

Interrupt is used for reception. Octets are buffered until the termination character reception. The frame is immediately analyzed and processed in interrupt function, except when analog to digital conversion is needed. For transmission, the frame is buffered and interrupt is enabled. After sending the termination character, the interrupt is disabled.

#### Impedance recording during tDCS (standalone mode)

When RS232 is enabled, the stimulator sends the impedance of the rodent during the stimulation via a serial port every 0.1 s. Frames are composed of a prefix, impedance value and unit:

- +XKR: + indicates fade-in, X is a value of impedance and KR for kΩ
*XKR: * indicates stimulation in progress
- #XKR: # indicates a pause during a stimulation
-XKR:—indicates fade-out

#### Computer-controlled mode

In this operating mode, the computer controls the set-point current and receives the feedback concerning voltage, current or current set-point. The following command must be used:

- IX: I to change the current set-point, X its value
- +: for one-step (0.25 μA) increase of the current set-point
-: for one-step (0.25 μA) decrease of the current set-point
- A: request to receive the value of the current set-point
- v: request to receive the voltage value across the rodent
- i: request to receive the current value through the rodent

For each request sent by the computer, the stimulator returns a hand-check, which consists of the same first character received except when an error occurred. In this case, the stimulator returns the type of error: EOFBR for an overfull of buffer (command is too long), ENOTCMD when the command is not correct or EOOL if the value is out of limit. A hand-check has to be performed before sending a new command.

In the case of command A, v or i, the return value is a raw data:

- A: value of PWM registers (10 bits)
- v and i: values of analog to digital conversion (10 bits)

This allows to leave computational time and to access to accurate measures. Concerning the command I, X is written in PWM registers. Indeed, the resolution is 0.25 μA.

Note that the recorded stimulation parameters such as impedance and voltage are available for offline analysis in the computer-controlled mode. In the standalone mode, the impedance and voltage are displayed on the LCD screen in real-time but are not recorded.

### Rounding error and approximation

#### Duration of fade-in and fade-out

The sample rate sets the duration of fade-in and fade-out: for each sample, the current increases or decreases in increments of 0.25 μA. The sample rate is calculated in the firmware as follows:

$$SampleRate=\frac{1000}{4\text{I}}\;Duration$$

$$SampleRate=\frac{250}{\text{I}}\;Duration$$

with *Sample Rate* in ms, *Duration* in s and I in μA.

**Fig 3:**
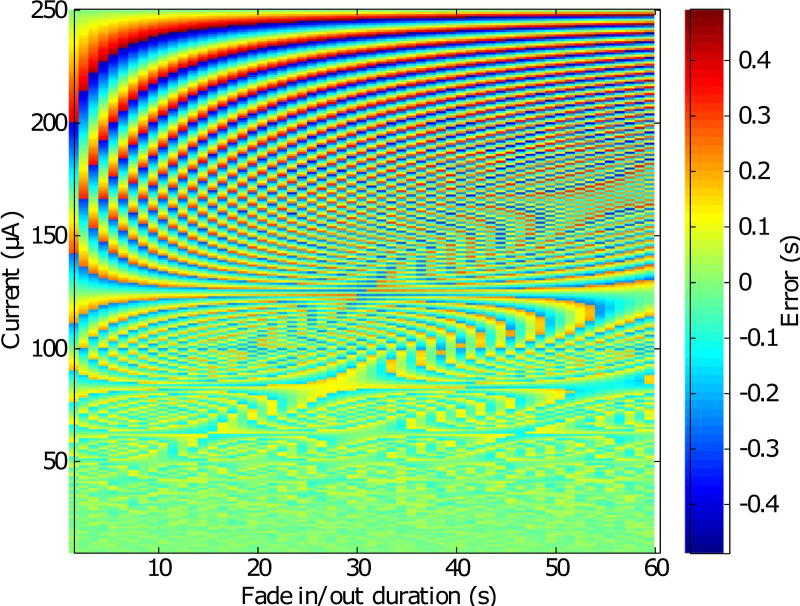
Fade-in/fade-out rounding error. The color plot shows the fade-in/fade-out rounding error according to its duration and current stimulation.

*SampleRate* is declared as an integer, inducing a rounding error. This error depends on the parameters of stimulation (fade-in/fade-out, current). Whatever the values set, the error does not exceed 0.5 s (Fig 3).

#### Voltage displayed

The relationship between the digital value measured by the A/D converter and the displayed voltage is:

$$Voltage=\frac{{\text{D}}_{\text{val}}V_{dd}}{1024\text{H}}$$

$$Voltage=0.293\;{\text{D}}_{\text{val}}$$

with *D*_*val*_ the digital value, *H* the ratio due to the differential amplifier (3.3) and *Voltage* the voltage across the rodent in decivolt. Floating-point type is not appropriated with this kind of microcontroller. That is why the approximate value of voltage is calculated as follows:

$$Voltage=\text{round}\left(\frac{2{\text{D}}_{\text{val}}}{7}\right)$$

**Fig 4:**
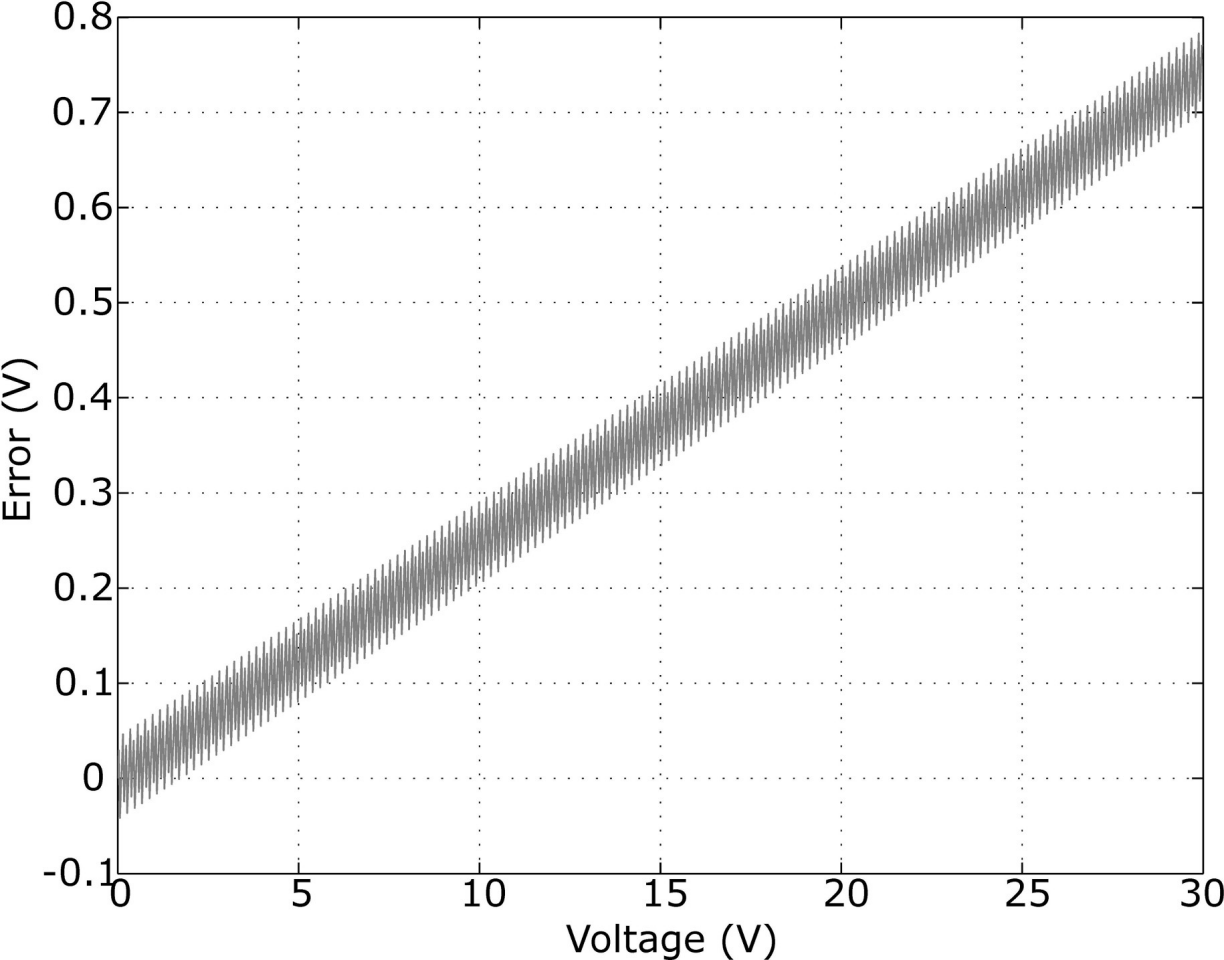
An error of voltage displayed on the stimulator. The rounded causes an error around 0.1 V and the approximation causes an error increasing with the voltage.

The approximation of 0.293 by $\frac{2}{7}$ and the rounded are the causes of an error plotted in Fig 4.

### Characterization

#### Response time and step response

The paradigm consists of a switching between two set-point values of 0 and 250 μA. We proceeded to 100 consecutive measures of rising edges. We defined the response time as a period between the request sending (from computer to stimulator) and the beginning of the current rising (over 0.1 μA). For the step response, we measured delays to obtain 99% and 99.9% of the set-point. The current was measured with a sample rate of 1 KHz (data acquisition system: MP36, BIOPAC Systems Inc., CA, USA). A computer program written in Matlab (MathWorks, USA) controlled the stimulator and synced the data acquisition.

#### Accuracy and precision

Precision and accuracy were assessed with measures for 26 current values ranging from 0 to 250 μA in increments of 10 μA. These measures (total: 5 s) were randomized and repeated 50 times. Only the last 0.5 s (steady state of the system) were considered. The current was acquired with a sample rate of 100 Hz (MP36, BIOPAC Systems Inc., CA, USA). A Matlab program drove the stimulator and synced it with the data acquisition system.

### Behavioral test on a rodent model

In an animal model, we have tested whether the Open-tES generator allows the reproduction of behavioral effects induced by a commercial tDCS stimulator (NeuroConn, Ilnemau, Germany, Table 1). We have previously demonstrated in mice with the NeuroConn generator that repeated anodal tDCS over the frontal lobe induces long-lasting modulation of the behavior, decreasing the depressive-like behaviors in the forced swim test (3 weeks after the end of the last anodal stimulations) [4]. Here, we compared our previous results in the forced swim test with those of a new group of mice stimulated with the Open-tES generator.

**Table 1:** Main technical features of the DC-Stimulator Plus (NeuroConn) and the Open-tES stimulator.

	Power supply	Mode	Current	Stimulation range	Max impedance allowed (user-set levels)	Pause/ resume stimulation easily	CE	Price 2020
**DC-Stimulator Plus (Clinical)**	Battery	Standalone	Direct, pulse, sinus wave, noise	0 to 4500 μA 25 μA increments	5–90 kΩ; 5 kΩ increments; Stimulation stops	No	Yes	~5000€
**Open-tES (Preclinical)**	Battery	Standalone or computer-controlled	Direct (Standalone mode) or programmable (computer-controlled mode)	10 to 250 μA, 1 or 0.25 μA increments (standalone or computer-controlled mode, respectively)	10–150 kΩ; 1 kΩ increments; warning alert: buzzer; User can pause the stimulation	Yes	No	~250€

#### Animals

Adult Swiss female mice (Janvier, Le Genest-Saint-Isle, France) were housed 10 per cage under standard laboratory conditions (12:12-hour light/dark cycle; lights on at 7:00 am) with food and water available *ad libitum*. Before the surgery, mice were allowed one week of acclimation, during which they were repeatedly handled. All procedures met the NIH guidelines for the care and use of laboratory animals and were approved by the University of Franche-Comté Animal Care and Use Committee (CEBEA-58).

#### Surgery and stimulation protocol

We conducted the same protocol as in our previous study [4,5]. Briefly, a tubular plastic jack (electrode holder, contact area: 3.5 mm^2^) was surgically fixed with a coating of glass ionomer cement (GC Fuji I, Leuven, Belgium) onto the skull of the anesthetized animals. The center of the electrode holder was positioned over the left frontal cortex 1 mm anterior to the coronal fissure and 1 mm left of the sagittal fissure. After surgery, all animals were allowed to recover for one week before undergoing tDCS. The electrode holder was then filled with a saline solution, and the stimulation electrode (DIXI Medical, Besançon, France) was attached. A larger conventional rubber-plate electrode (cathode, 9.5 cm^2^; Physiomed Elektromedizin AG, Schnaittach, Germany) placed onto the ventral thorax served as the counter electrode. An anodal 2 x 20 min/day (5-hour interstimulation interval) constant current of 200 μA was applied transcranially over the frontal cortex using the Open-tES device or the commercial device (DC-Stimulator Plus NeuroConn, Germany) for five consecutive days, with a linear fade-in/fade-out of 10 s. Animals were awake and restrained during the tDCS (see [4]); control animals were subjected to the same procedure (surgeries, restraint box, electrode montage), but the current was not delivered. Animals received the tDCS in the morning (9:00 am-12:00 pm) and in the afternoon (2:00 pm-5:00 pm) during the light phase of the cycle (light on: 7:00 am; light off: 7:00 pm).

#### Forced swim test

The forced swim test, adapted from Porsolt and colleagues [35], is the most common tool used to evaluate the antidepressant efficacy of new treatments in a rodent. Mice are placed into a beaker (height: 26 cm, diameter: 18 cm) containing water at a temperature of 30 ± 3°C and a depth of 17 cm, so that they could neither escape nor touch the bottom. Each test lasts 6 min and is video-recorded for subsequent scoring by a blind observer of the total time spent immobile (immobility of the mice in the water). Mice are considered immobile when they cease struggling and remain floating motionless in the water for at least 2 seconds. A high score of immobility is associated with a depressive-like behavior in rodents. To reproduce the effects observed in our previous study with a commercial stimulator [4], we evaluated the time of immobility three weeks after the last stimulation. The forced swim test was performed in the morning (9:00 am-12:00 pm) during the light phase of the cycle.

#### Statistical analysis

The results are expressed as mean ± SEM. Significance is set at p < 0.05. One-way analysis of variance (ANOVA) was used to analyze depression-related behavior (reflected by the time of immobility of the mice during the test) with the group as between-subject variable (sham N = 13, NeurConn N = 8, Open-tES N = 8). Newman-Keuls post-hoc tests were used to describe differences between individual groups.

## Results

### Response time and step response

**Fig 5:**
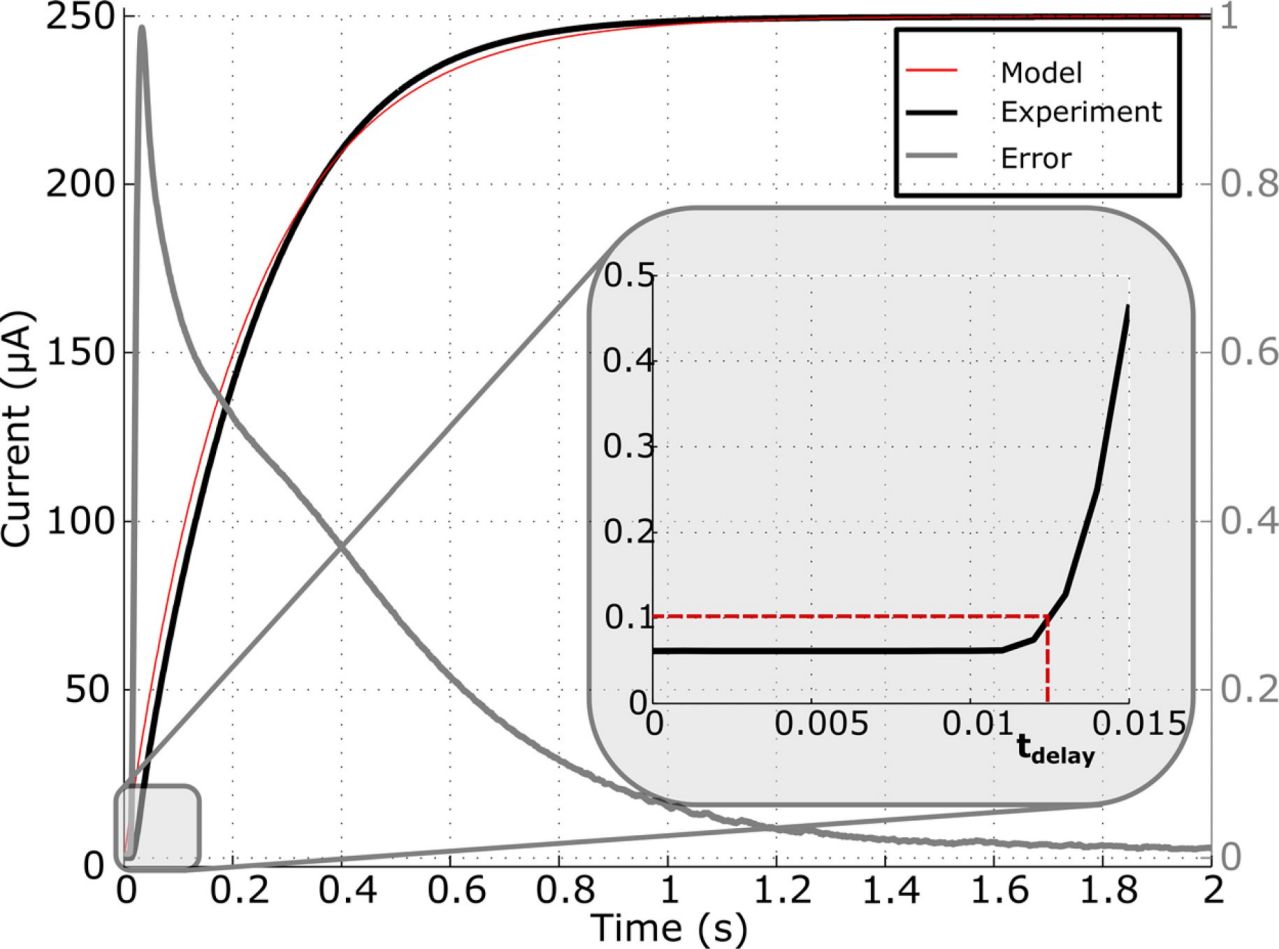
Step response of the system. It is a typical step response for a first-order system with a delay t_delay_ due to RS232 communication.

The average of the response time is 14.21 ± 0.9022 ms (t_delay_ Fig 5). The step response is typically a first-order resistor-capacitor filter. Therefore, the equation is:

$$I\left(t\right)=\left({\text{I}}_{\text{init}}-{\text{I}}_{\text{setpoint}}\right)\text{exp}\left(-\frac{1}{\tau }\right)$$

with I_init_ the current before the new set-point, I_setpoint_ the new set-point and τ = RC the time constant. Differences between the model and the experiment curve are mainly due to the t_delay_, which is not taken into account in the equation (Fig 5). In these measurement conditions (I_init_ = 0 μA and I_setpoint_ = 250 μA), the delay is 909 ± 60 ms to reach 99% and 1580.58 ± 38.53 ms for 99.9% of the set-point value.

### Accuracy and precision

**Fig 6:**
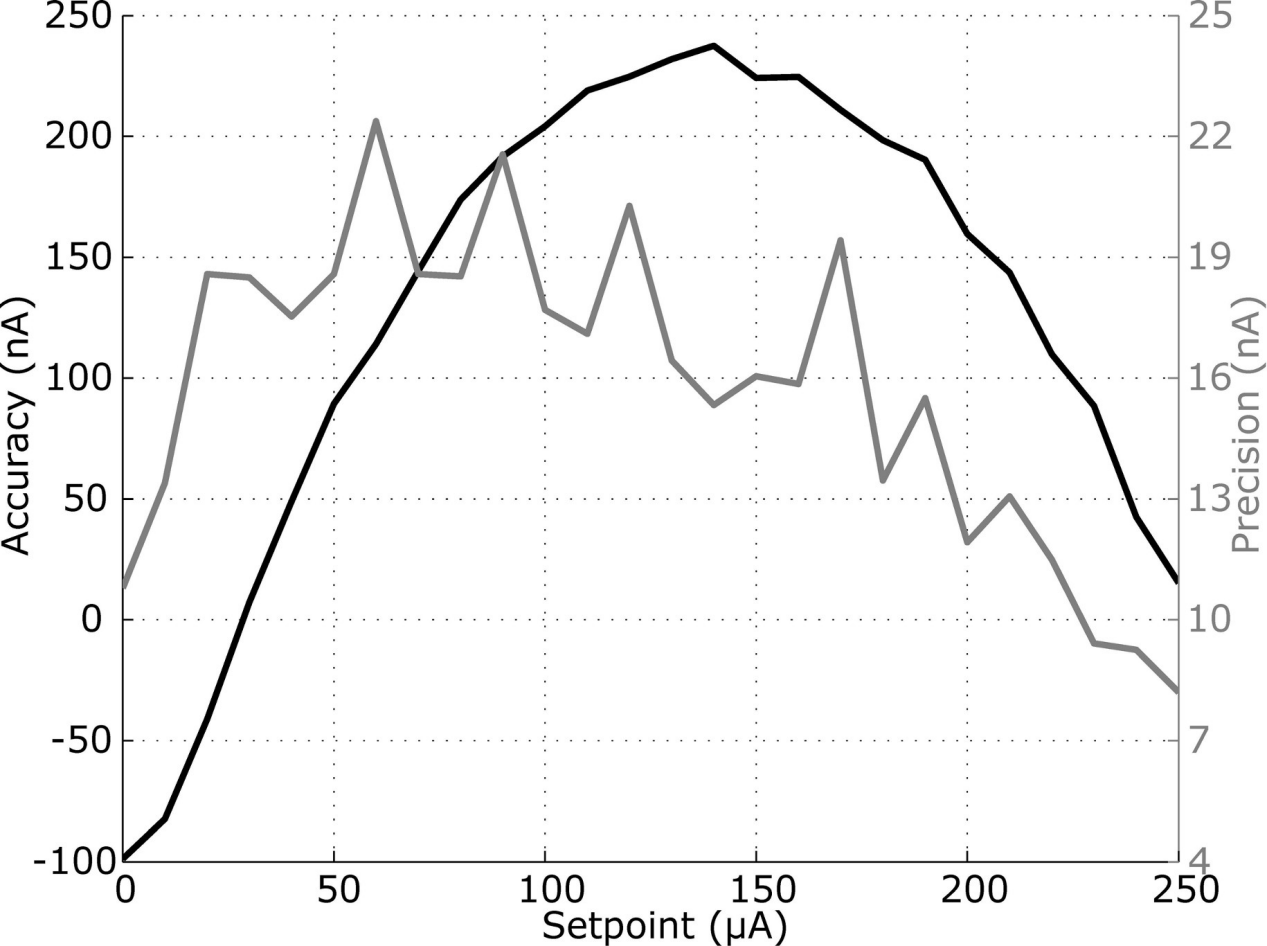
Precision and accuracy of the stimulator.

For the set-point from 0 to 140 μA, the accuracy rises from -99 nA to 237 nA. Passing the set-point of 140 μA, it goes down to 15 nA (Fig 6). The precision is up to 22 nA for a current set-point of 70 μA and down to 8 nA for a current set-point of 250 μA.

### Forced swim test

**Fig 7:**
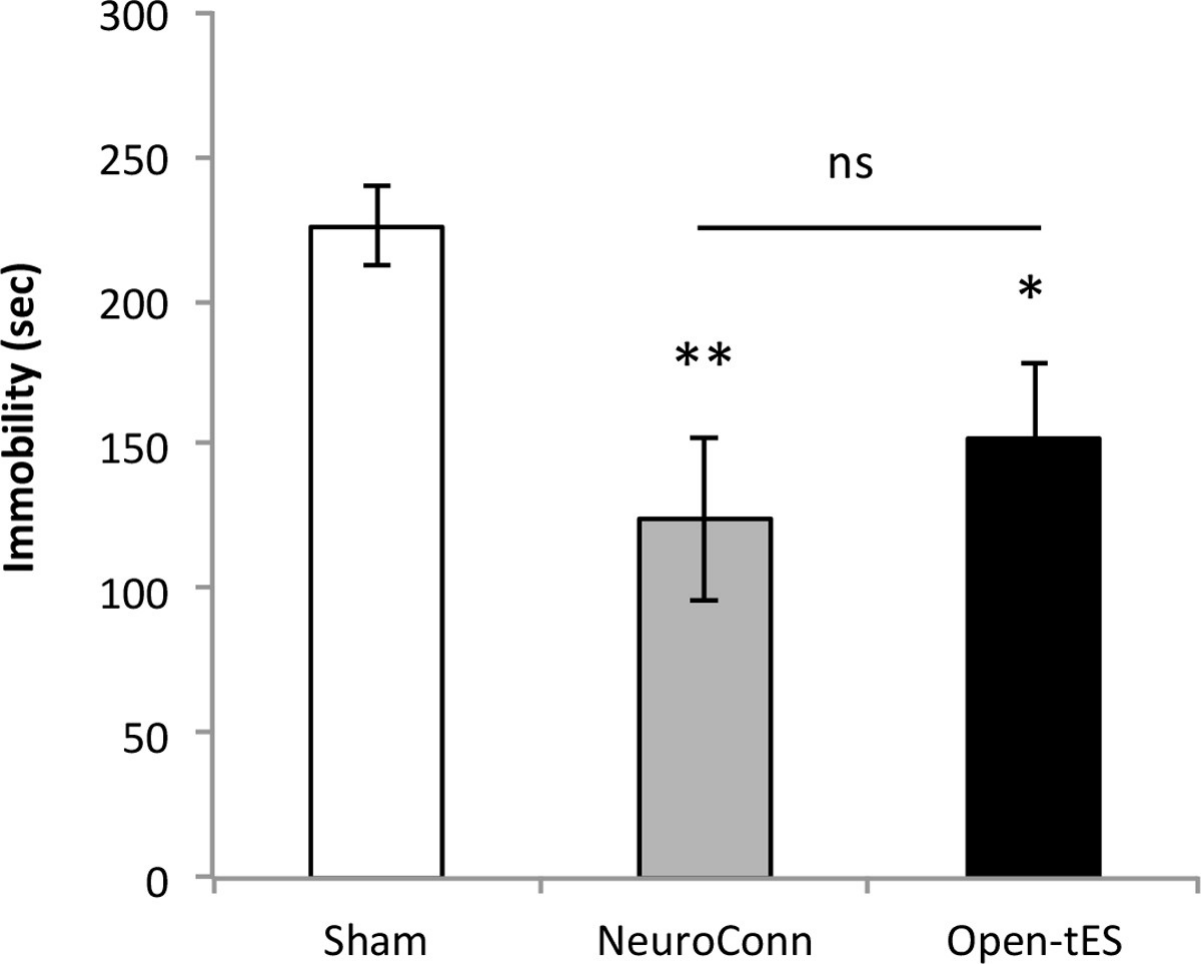
Forced swim test. Eight-week-old Swiss female mice were subjected to repeated anodal tDCS with the NeuroConn or Open-tES generator, for five consecutive days (2 x 20 min/day constant current, 200 μA). Depressive-like behavior was tested three weeks after the last electrical stimulation (sham N = 13, NeuroConn generator N = 8, Open-tES generator N = 8). *p < 0.05 and **p < 0.01 versus sham, ns: not significant.

In Fig 7, we present results obtained with the Open-tES generator and compared them to those previously obtained with a commercial stimulator (DC-Stimulator Plus NeuroConn, Germany). tDCS has a significant effect on the time of immobility in the forced swim test 3 weeks after the last stimulation (ANOVA treatment: F_(2; 26)_ = 6.71, p < 0.01). Post-hoc Newman-Keuls analysis revealed that, relative to sham controls, stimulations with either the NeuroConn or the Open-tES generator significantly decreased the time the animals spent immobile (p < 0.01 and p < 0.05, respectively). There was no significant difference between the effects of the Open-tES and the NeuroConn stimulators on the time of immobility. Therefore, the repeated anodal stimulations with the Open-tES and the NeuroConn stimulator induced similar antidepressant effect three weeks after the end of the stimulations.

## Discussion

Open-tES device induces comparable effects in a commonly used behavioral task as those induced by a commercially available current generator (DC-Stimulator Plus NeuroConn, Germany). Considering its multi-functionality, its current ranging from 0 to 250 μA in increments up to 0.25 μA and its precision, reliability, and accuracy, our generator appears to be highly adapted to preclinical research. Low-cost (approximately 250 euros without components assembly) and easy-to-use, the Open-tES allows the tDCS to be applied with customized parameters in the stand-alone mode. This version offers new features, e.g. the possibility to record stimulation parameters (such as impedance) in the controlled-computer mode. Being able to pause and restart the stimulation offers the possibility to solve any problems that may be encountered during the stimulation. With the computer-controlled mode, the user has considerable freedom of customization (otDCS, tPCS or arbitrary waveform). For example, the user can sync or trigger the stimulation with external parameters (e.g. EEG). Some MatLab scripts are available from the repository to ease the programming. We initially developed this device to meet our current needs, but certain technical specifications can be easily adjusted. For example, the current ranging can be expanded by changing the R5 resistor value (Fig 2). The response time and step response are suitable for the most common applications, but if faster response time is needed it is possible to increase RS232 baud rate (it ensures the communication between the stimulator and the computer). Another way to do it is to raise the cut-off filter changing R FILT and C FILT (Fig 2) if the electromagnetic environment is favorable.

Nevertheless, PWM frequency has to be attenuated by the filter. Sharing the project source ensures a straightforward reproduction of the simulator. Our future direction is to offer substantial improvements to the device, allowing it to send a negative current. That will allow the realization of other kinds of electrical stimulation (as tACS or tRNS) and will increase its flexibility.
